# Cerebrospinal Fluid Inflammatory Biomarkers Reflect Clinical Severity in Huntington’s Disease

**DOI:** 10.1371/journal.pone.0163479

**Published:** 2016-09-22

**Authors:** Filipe Brogueira Rodrigues, Lauren M. Byrne, Peter McColgan, Nicola Robertson, Sarah J. Tabrizi, Henrik Zetterberg, Edward J. Wild

**Affiliations:** 1 Huntington’s Disease Centre, Institute of Neurology, University College London, London, United Kingdom; 2 Institute of Neuroscience and Physiology, Department of Psychiatry and Neurochemistry, the Sahlgrenska Academy at the University of Gothenburg, Mölndal, Sweden; 3 Department of Molecular Neuroscience, Institute of Neurology, University College London, London, United Kingdom; Centre de Recherche Jean-Pierre Aubert, FRANCE

## Abstract

**Introduction:**

Immune system activation is involved in Huntington’s disease (HD) pathogenesis and biomarkers for this process could be relevant to study the disease and characterise the therapeutic response to specific interventions. We aimed to study inflammatory cytokines and microglial markers in the CSF of HD patients.

**Methods:**

CSF TNF-α, IL-1β, IL-6, IL-8, YKL-40, chitotriosidase, total tau and neurofilament light chain (NFL) from 23 mutation carriers and 14 healthy controls were assayed.

**Results:**

CSF TNF-α and IL-1β were below the limit of detection. Mutation carriers had higher YKL-40 (p = 0.003), chitotriosidase (p = 0.015) and IL-6 (p = 0.041) than controls. YKL-40 significantly correlated with disease stage (p = 0.007), UHDRS total functional capacity score (r = -0.46, p = 0.016), and UHDRS total motor score (r = 0.59, p = 4.5*10^−4^) after adjustment for age.

**Conclusion:**

YKL-40 levels in CSF may, after further study, come to have a role as biomarkers for some aspects of HD. Further investigation is needed to support our exploratory findings.

## Introduction

Huntington’s disease (HD) is a neurodegenerative condition in which progressive decline in cognitive, motor and behaviour functions are a consequence of neuronal dysfunction and death. The primary cause of HD is known–a polyglutamine expansion in the first exon of the *HTT* gene[1, 2]–but the mechanisms by which mutant huntingtin (mHTT) protein leads to neuronal cell death still need clarification. The immune system, both peripherally and in the central nervous system (CNS), has been implicated in HD pathogenesis[3, 4]. Numerous *post-mortem* and *in vivo* studies have shown that complement activation[5], microglial activation[6],[7] and concentrations of pro-inflammatory and immunomodulatory cytokines IL-1β, IL-6, IL-8, IL-10, CCl2 and TNF-α[4, 8] are increased in peripheral blood in HD patients compared with controls. Animal studies suggest that inhibition of inflammatory pathways could ameliorate HD severity[9, 10]. One compound aimed at modulating inflammatory glial activity, laquinimod, is currently being tested in HD and several other clinical trials are being prepared.[11, 12]

Biomarkers reflecting these peripheral and/or central derangements of neuroinflammation could be useful to better characterise disease progression and the therapeutic response to specific interventions. Cerebrospinal fluid (CSF) is a relatively accessible body fluid, enriched in brain-derived proteins[13], that has proven to be of value as a source of biomarkers in HD[14] and other neurodegenerative diseases[4, 15, 16]. In 2014, Vinther-Jensen and colleagues found a statistically non-significant trend towards increasing CSF levels of the microglial marker YKL-40 with later disease stage, but no association with disease severity or replication has been sought in HD CSF collected under strictly standardised conditions.[17]

In this work, we explored immune-associated substances previously reported to be altered in CSF or plasma in HD, namely proinflammatory cytokines–TNF-α, IL-1β, IL-6 and IL-8 –and microglial markers–YKL-40 and chitotriosidase–in the CSF of well-characterised patients, to determine what markers are capable of predicting clinical severity in HD. By measuring total tau and neurofilament light chain (NFL)–established markers of neuronal cell death[18, 19]–in the same CSF samples, we also examined whether neuroinflammation and neurodegeneration are linked.

## Materials and Methods

Ethical approval was given by the joint University College London/ University College London Hospitals ethics committee. All patients gave informed written consent before enrolment. Patient consent, inclusion and exclusion criteria, clinical assessment, CSF collection and storage were as previously published.[14] In brief, samples were collected after an overnight fast at the same time of day and centrifuged and aliquoted rapidly on ice using a standardised protocol and polypropylene plasticware.[14] Healthy controls were contemporaneously recruited, drawn from a population with a similar age to patients, and clinically well, so the risk of incidental neurodegenerative diseases was very low. Relevant aspects of clinical severity were quantified using the total functional capacity (TFC) and total motor score (TMS) components of the Unified Huntington’s Disease Rating Scale (UHDRS).[20] Disease burden score, a function of age and CAG repeat length known to predict many features of HD onset and progression, was calculated. [21, 22]

### CSF analyte quantification

CSF TNF-α, IL-1β, IL-6 and IL-8 were measured using a Meso Scale Discovery antibody-based tetra-plex array with electrochemiluminiscence detection according to the manufacturer’s instructions (Meso Scale Discovery, Gaithersburg, MD, USA). CSF TNF-α and IL-1β concentrations were below the lower limits of detection (LODs) of the assay for all samples. CSF IL-6 and IL-8 concentrations were above the LODs (0.32 pg/mL and 0.25 pg/mL, respectively) in all samples, except for one in which IL-6 was <0.32 pg/mL. CSF YKL-40 concentration was measured by enzyme-linked immunosorbent assay (ELISA) according to the manufacturer’s instructions (R&D Systems Inc. Minneapolis, MN). This assay has an LOD of 6.25 ng/mL and all samples were well above this limit.

CSF chitotriosidase was measured using an in-house enzyme activity assay, as previously described.[23] Two samples in the HD group and eight samples in the control group were below the LOD of the assay (0.2 nkat/L). This distribution of undetectable values was statistically significant between the two groups (Chi-square 10.3569, p = 0.00129), in keeping with a disease-related difference (see results section). CSF total tau was quantified using the INNOTEST enzyme-linked immunosorbent assay (ELISA) according to the manufacturer’s instructions (Fujirebio, Ghent, Belgium). CSF NFL was measured using the NF-light ELISA according to the manufacturer’s instructions (UmanDiagnostics, Umeå, Sweden). All samples had CSF total tau and NFL concentrations above the LODs for the assays (45.8 and 50 ng/L, respectively). All measurements were performed as single analyses in one round of experiments using one batch of reagents. Based on internal quality control samples intra-assay coefficients of variation were 30% for IL-6, 12% for IL-8, 7% for YKL-40, 10% for chitotriosidase, 10% for total tau and 15% for NFL. Laboratory technicians were board-certified and blinded to clinical data.

### Statistical analysis

Statistical analysis was performed with Stata 14 software (StataCorp, TX, USA). We used one-way ANOVA to assess baseline intergroup differences. Potentially confounding demographic variables (age, gender) were examined in preliminary analyses and those found significant were included as covariates for subsequent analyses. Samples below the LOD were assigned the LOD concentration. The distributions of all tested molecules were tested for normality. Two-group comparisons were tested using unpaired two-tailed t-test or Wilcoxon log-rank test. Stepwise forward logistic regression analysis was used to find combinations of molecules with better diagnostic performance, and these combinations’ receiver operating characteristics were compared with the best molecule alone. To test associations with disease progression we calculated Pearson’s and partial correlations coefficients. Where appropriate, bootstrapping with 1,000 repetitions was applied to non-normal variables. Significance level was defined as p<0.05. Where there was concern about a single participant group or outliers unduly influencing the analysis, a sensitivity analysis was conducted, repeating the tests with those datapoints excluded.

## Results

### Participants’ characteristics

Thirty-seven cross-sectional CSF samples were obtained from 14 healthy controls, 3 pre-manifest gene expansion carriers (HDGECs), and 20 manifest HDGECs. Details are given in Table 1. There was no significant difference in age or gender distribution among the included groups. CAG repeat number did not vary significantly between HDGEC groups.

**Table 1:** Characteristics of included participants by disease stage.

	n (%)	Age (±SD)	M:F ratio	CAG	TFC	TMS	Disease burden
**Total sample**	**37**	**47 (±12)**	**14:23**				
Healthy controls	14 (38)	44 (±13)	4:10	N/A	13	N/A	N/A
Pre-manifest	3 (8)	40 (±10)	1:2	42	13	1	252
Early stage	15 (41)	50 (±12)	6:9	44	11	27	407
Moderate stage	5 (14)	58 (±2)	3:2	43	5	50	421
*Intergroup differences*		*p = 0*.*077*	*p = 0*.*716*	*p = 0*.*261*	p = 1*10^*−8*^	*p = 0*.*005*	*p = 0*.*007*

M:F, male to female ratio; CAG, Cytosine-adenosine-guanine repeats; TFC, total functional score; TMS, total motor score; N/A, not applicable.

### CSF concentrations

Medians and interquartile range (IQR) of CSF concentrations of quantified substances are shown in Table 2. Only IL-8 was normally distributed. CSF TNF-α and IL-1β concentrations were below the limit of detection in all cases. No substance varied between genders. YKL-40 and IL-8 were significantly correlated with age in healthy control participants (r = 0.82, p = 2.6*10^−12^ for YKL-40 and r = 0.50, p = 0.002 for IL-8) so age was used as a covariate for these analyses. No other associations with age were found.

**Table 2:** CSF biomarkers concentrations by disease stage.

	YKL-40ng/mL(median, IQR)	Chitotriosidasenkat/L(median, IQR)	IL-6pg/mL(median, IQR)	IL-8pg/mL(median, IQR)	NfLpg/mL(median, IQR)	taupg/mL(median, IQR)
**Total**	**116.76 (103.43)**	**0.47 (0.46)**	**0.85 (0.40)**	**33.50 (7.50)**	**1223 (1625)**	**238 (105)**
Healthy controls	83.071 (58.133)	0.20 (0.27)	0.70 (0.30)	34.00 (19.00)	300 (134)	192 (104)
Pre-manifest	91.420 (54.687)	0.47 (0.32)	0.90 (0.40)	28.00 (11.00)	842 (365)	196 (87)
Early stage	124.757 (95.458)	0.51 (0.34)	1.00 (0.80)	33.00 (5.00)	1969 (1126)	252 (126)
Moderate stage	194.121 (16.371)	0.41 (0.82)	0.95 (0.80)	38.00 (10.50)	2231 (994)	384 (183)

IQR, interquartile range; IL, interleukin; NfL, neurofilament light chain.

### Effects of haemoglobin contamination

The concentration of CSF haemoglobin was not found to be associated with the concentrations of any analytes, and therefore we assume any minimal blood contamination did not interfere with quantification of the analytes.

### Comparison between healthy controls versus gene expansion carriers

**Fig 1:**
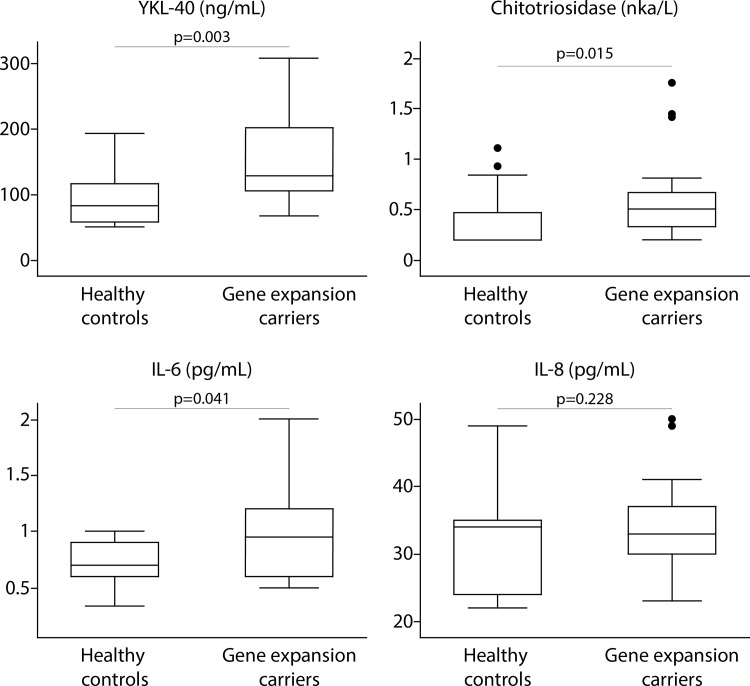
Comparison in CSF levels of inflammatory molecules between 14 healthy controls versus 23 gene expansion carriers. The p-values for unpaired two-tailed t-test (Il-8) or Wilcoxon log-rank test (YKL-40, chitotriosidase, IL-6) are shown.

CSF YKL-40, chitotriosidase and IL-6 were significantly elevated in HDGECs compared with controls (Fig 1; p = 0.003, AUC = 0.797 for YKL-40, p = 0.015, AUC = 0.7419 for chitotriosidase; p = 0.041, AUC = 0.7029 for IL-6). IL-8 was not significantly altered between these groups (p = 0.228, AUC = 0.5909). Forward stepwise logistic regression analysis showed that the combination of YKL40 and IL-6 had a higher diagnostic power (AUC = 0.8620) than any of the individual molecules. However, this combination was not statistically significantly better than YKL-40 alone (p = 0.2674).

### Associations with disease stage

**Fig 2:**
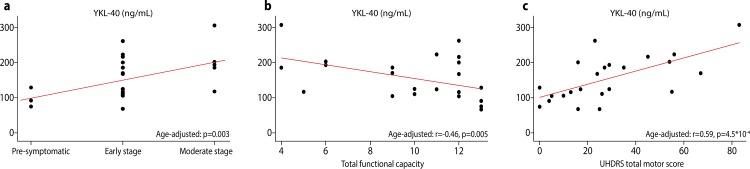
Relationship between CSF concentration of YKL-40 and measures of disease progression and clinical severity. a, disease stage; b, UHDRS total functional score; c, UHDRS total motor score. The p-values for partial Pearson’s correlation coefficient adjusted for age are shown.

In HDGECs, there was a statistically significant positive association between CSF YKL-40 level and disease stage (p = 0.001, Fig 2A). This association remained significant after controlling for age (p = 0.003). Repeating the analysis excluding premanifest individuals (p = 0.018), outliers (p = 0.005) and both (p = 0.013) supported the statistical significance of this association. CSF IL-8 level, too, was significantly positively associated with disease stage (p = 0.023), but not after controlling for age (p = 0.153). Chitotriosidase and IL-6 were not significantly associated with disease stage (p = 0.891 and p = 0.398, respectively).

### Associations with clinical severity

There was a statistically significant negative association between CSF YKL-40 and TFC after controlling for age (r = -0.46, p = 0.005; Fig 2B), but not disease burden score (r = -0.36, p = 0.090). CSF chitotriosidase, IL-6 and IL-8 were not significantly associated with TFC (r = 0.12, p = 0.607; r = -0.16, p = 0. 531 and r = -0.37, p = 0.093, respectively).

CSF YKL-40 was significantly positively associated with TMS (r = 0.65, p = 1.3*10^−5^, Fig 2C). This association remained significant after controlling for age (r = 0.59, p = 4.5*10^−4^), and also after controlling for disease burden score (r = 0.49, p = 0.037), indicating that CSF YKL-40 can independently predict severity of motor manifestations beyond the known predictive value of age and CAG repeat length. Chitotriosidase, IL-6 and IL-8 were not statistically significantly associated with TMS (r = -0.15, p = 0.234; r = -0.08, p = 0.666; and r = 0.23, p = 0.3055, respectively).

### Associations with markers of neuronal death

Because they had been found to be associated with measures of clinical severity, YKL-40 and IL-6 were further examined for association with CSF tau and NFL levels, each of which has been shown to be associated with neuronal death in neurodegenerative and other conditions.[24–26] There were statistically significant positive associations between YKL-40 and both total tau (r = 0.65,.p = 1.4*10^−5^) and NFL (r = 0.72,.p = 3.7*10^−26^), which remained significant after controlling for age (r = 0.69, p = 1.2*10^−4^; r = 0.72, p = 1.5*10^−9^, respectively), and disease burden score (r = 0.51, p = 0.006; r = 0.55, p = 3.5*10^−6^, respectively), again suggesting that YKL-40 level can predict these markers of neuronal death, independent of their mutual association with age and CAG repeat length. IL-6 did not show any such association with tau or NFL (r = 0.17, p = 0.559; r = 0,23, p = 0.300, respectively).

## Discussion

In this exploratory cross-sectional study using CSF samples collected and processed under rigorously controlled conditions, we show that CSF concentrations of the microglial markers YKL-40 and chitotriosidase, and the pro-inflammatory cytokine IL-6, were elevated in HDGECs compared with healthy controls.

YKL-40 was most robustly associated with clinical severity: age-adjusted YKL-40 CSF concentrations in gene expansion carriers were associated with disease stage and with clinical measurements of disease progression—UHDRS TFC and UHDRS TMS. These latter associations remained statistically significant after adjustment for disease burden score, indicating that CSF YKL-40 has independent clinical predictive power, beyond its association with age and CAG repeat length. To our knowledge, the only other CSF substance for which such independent predictive power has been reported is the pathological agent itself, mutant huntingtin protein (mHTT).[14]

Furthermore, CSF YKL-40 was associated with the CSF markers of neuronal death tau and NFL; and retained this predictive power after adjustment for the mutually-associated predictors age and CAG repeat length.

YKL-40, also known as chitinase 3-like protein 1 (CHI3L1), is a member of the 18 glycosyl hydrolase family, without enzymatic activity and with poorly understood function.[27] During neuroinflammation YKL-40 is expressed and secreted by microglia[28] and is associated with astrocytosis and astrocytic motility.[29] Neuroinflammation is thought to have an important role on HD pathogenesis and mHTT has a direct effect on the NFκB pathway.[30] This interaction induces expression of pro-inflammatory molecules and may explain the elevation of YKL-40 in HD CSF.

IL-6 in CSF–previously shown to be elevated in HD blood plasma–was found to be associated with disease stage but not with more granular clinical measures of severity or neuronal death. IL-6 is known to cross the blood-brain barrier[31] so it seems most likely that its CSF level reflects systemic elevation or possibly parallel CNS and peripheral immune activation.[4] Nevertheless these results should be interpreted with caution since the coefficient of variation of this assay was relatively high.

To our knowledge, this is the first study describing the potential of CSF YKL-40 as an independent predictor of clinical severity and neuronal death in HD. Our findings differ from those of Vinther-Jensen et al, who did not find a statistically significant association between CSF YKL-40 and disease stage or severity.[17] Differences in sample collection, processing and assay methodology may explain the differences in our findings. Our standardised protocols aim to reduce avoidable inter-subject variability by controlling fasting state, time of day, collection methodology, processing methods and equipment. Still, it would be sensible to further validate these differences in larger cohorts. To our knowledge, our study is the first to explore the relationships between CSF inflammatory markers and clinical variables such as TFC and TMS, and biomarkers of neuronal cell death.[17]

Notwithstanding the striking findings in respect of YKL-40, this exploratory study requires validation in larger studies such as the HDClarity initiative.[32] The longitudinal predictive power of CSF YKL-40 should be examined in studies with repeat CSF collection. Furthermore, evaluation in blood of biomarkers proposed from CSF is an important focus of our work. Blood has hitherto failed to yield meaningful biomarkers of CNS disease activity but this remains an important aim if suitably CNS-specific blood markers can be identified.[33]

## Conclusion

We conclude that IL-6, chitotriosidase and YKL-40 show disease-related elevations in CSF in HD, affirming the role of microglial activation and the innate immune system in the disease. CSF YKL-40 in particular can independently predict clinical severity and neuronal death, and may be a useful targeted biomarker for the contribution of microglial dysfunction to disease activity.

## Supporting Information

S1 Appendix Dataset. (XLSX)
